# Joint Efforts Towards Capacity Building in International and Global Health

**DOI:** 10.3389/phrs.2022.1604688

**Published:** 2022-01-27

**Authors:** Axel Hoffmann

**Affiliations:** ^1^ Swiss Tropical and Public Health Institute, Basel, Switzerland; ^2^ University of Basel, Switzerland

**Keywords:** capacity building, training programs, quality assurance, transnational collaboration, long-term partnership

Academic capacity building of health personnel in international and global health, based on scientific evidence, is one of the pillars of the global strategy on human resources for health (HRH) [1]. Academic partnerships often focus on supporting institutions in single low-and middle-income countries (LMIC), successfully shown with the large-scale program for Rwanda [2], or establishing a multidisciplinary, international PhD network for training future researchers in Africa [3].

With the aim to establish a transnational Master program in Global/International Health, tropEd, an international training network exists since 25 years. With an initial funding under the umbrella of the European Erasmus program und exclusively with European universities as members, the network rapidly increased the number of members of non-European universities. Today the network—formally an association under German law since 2003—consists of 27 institutions of higher education from Africa, Asia, Europe and Latin America^1^. Whereas in the first years the mobility of students and teaching staff was prioritised, nowadays the exchange of experiences, a common standard of education in global health, and the quality assurance of teaching offers are in the focus.

Reflecting on the 25th anniversary of the network, the history of tropEd gives an excellent example, that long-term partnerships and collaborations of dedicated individuals and institutions are the key for successfully contributing to capacity building on the individual, but also at the institutional level. Students completing a Master’s program at one of the member institutions value the new knowledge and skills acquired as essential and for many of them the program laid the ground for job promotion [4]. In addition, the majority of students from LMICs remains in their country of origin after graduation, supporting their health systems in the daily fight against manifold challenges.

As students register at one of the member universities, but can take courses at any member institution, the quality assurance of offered courses is seen as one major task of the network. tropEd does not deliver the Master degree itself, therefore the network defined a framework with common minimum academic and quality assurance structures, content and criteria [5]. The national accredited degree of the respective member university must adhere to these standards to be recognized as a “tropEd Master in International Health” [5]. In addition, such a tropEd recognition will be delivered to a student only, if she/he took at least courses worth ten ECTS credit points outside the home institution.

As a first step of the institutional quality assurance, institutions applying for membership have to undergo a formalized self-evaluation, followed by a site visit of a member of tropEd’s Executive Committee, including discussions with students, staff and management of the applying institution; the final decision on admission is done by the General Assembly. The offered Master program has to be offered in a modular format, consisting of a core course, advanced modules and a Master thesis. Core course and advanced modules have to be submitted to the General assembly in a standardized format, and will be peer-evaluated and accredited for a maximum of 5 years; after this period courses have to be re-submitted and re-accredited. Only courses, which are accredited by tropEd, can be taken by the students. This strictly implemented peer-review process of courses is the only practical measure for guaranteeing the quality of course offers, as no accreditation agency for a worldwide transnational higher education network exists [5]. Although this quality assurance measure means an additional burden for the member institutions, it has proven its validity over the past 25 years.

Currently the SARS-CoV-2 pandemic forces the next challenges to tropEd: mobility of students and teachers was heavily restricted in the past 2 years, and national regulations often did not allow onsite teaching—as most of the universities worldwide also tropEd members have to change the mode of delivery of courses to an online or hybrid format. Online teaching does need different pedagogical approaches [6], what has to be taken into account into the quality assurance of the offered courses. Furthermore, tropEd will have to support as a network the efforts of decolonizing International and Global Health training [7], by increasing the number of member institutions from the global South, but also by a bigger share of courses offered from institutions from LMIC.

## Footnotes

1
https://troped.org/member-institutions-3/.
